# Radio-protective effect and mechanism of 4-Acetamido-2,2,6,6- tetramethylpiperidin-1-oxyl in HUVEC cells

**DOI:** 10.1186/s12199-017-0616-9

**Published:** 2017-03-24

**Authors:** Feng Wang, Peng Gao, Ling Guo, Ping Meng, Yuexing Fan, Yongbin Chen, Yanyun Lin, Guozhen Guo, Guirong Ding, Haibo Wang

**Affiliations:** 1 School of Preventive Medicine, Fourth Military Medical University, Xi’an, 710032 People’s Republic of China; 2 School of Pharmacy, Fourth Military Medical University, Xi’an, 710032 People’s Republic of China; 3 Shanxi Province Corps Hospital, Chinese People’s Armed Police Forces, Taiyuan, 030006 People’s Republic of China; 4 Department of urology, Xijing Hospital, Fourth Military Medical University, Xi’an, 710032 People’s Republic of China

**Keywords:** HUVEC cells, Ionizing radiation, Antioxidative, Radioprotection, Tempol

## Abstract

**Objectives:**

To search for more effective radiation protectors with minimal toxicity, a water-soluble nitroxides Acetamido-Tempol (AA-Tempol) was evaluated for potential radioprotective properties in HUVEC cells (Human Umbilical Vein Endothelial cell line).

**Methods:**

To study the anti-radiation effect of AA-Tempol in cell culture, the viability of irradiated HUVEC cells using a clonogenic survival assay was examined. The anti-apoptosis effects of AA-Tempol using Annexin V/propidium iodide staining in a flow cytometry assay was also evaluated. To elucidate the molecular mechanism of the anti-apoptosis effect of AA-Tempol against X-radiation induced HUVEC cell apoptosis, the expression of Bax, Bcl-2 and p53 and caspase-3 were examined. The changes in the level of malondialdehyde (MDA) and glutathione (GSH) in HUVEC cells after X-radiation were also investigated.

**Results:**

Pretreatment of the HUVEC cells colony with AA-Tempol 1 h before X-radiation significantly increased the colony survival (*p* < 0.05) compared with the cells without pretreatment. This demonstrates that AA-Tempol provides an effective radiation protection in the irradiated HUVEC cells, thus reducing apoptosis from 20.1 ± 1.3% in 8 Gy X-radiated cells to 12.2 ± 0.9% (1.0 mmol/L^−1^ AA-Tempol) in AA-Tempo pretreated HUVEC cells. This implies that 1.0 mM AA-Tempol treatment significantly block the increase of caspase-3 activity in radiated HUVEC cells (*P <* 0.01), causing down-regulation in expressions of Bax and P53 and up-regulation in the expression of Bcl-2. Pretreatment with AA-Tempol also decreased the MDA activities (*P <* 0.01) and increase the GSH level (*P <* 0.05) in HUVEC cells compared to the 8Gy X-radiated cells without pretreatment.

**Conclusions:**

These observations indicate that AA-Tempol is a potential therapeutic agent against the radiation damage.

## Introduction

With the advent of new technologies, ionizing radiation (X- and gamma-rays) has been widely used in different fields during the last decades, both in every branch of medicine, to diagnostic and diseases treatment; and in several industries,where human exposure to ionizing radiation has increased tremendously [1, 2]. Ionizing radiation causes damage to living tissues through a series of molecular events depending on the radiation energy. Its damaging effects and the intensity on human health have been reported [3]. Due to the hazardous effects of the ionizing radiation, the ability of some chemical compounds to protect cells from damaging effects of ionizing radiation has been reported. For instance, Patt et al. [4] made initial discovery about the protective effects of cysteine on the X-irradiation damage in mice. Other new agents, such as amifostine (WR2721) has been discovered and clinically approved by the Food and Drug Administration for reducing the side effects of radiotherapy in patients [5]. Several studies have also demonstrated that amifostine protects normal tissue from both acute and late radiation damage without protecting the tumor. In another term amifostine is a selective cytoprotector of normal tissues [6–9]. However, WR2721 has some side-effects such as hypotension, nausea, and vomiting, which are significantly augmented upon intravenous administration [10–12]. Hence, the development of effective and less toxic radioprotection agents is of great importance [13].

The biological effects of ionizing radiation are linked to the production of reactive oxygen species (ROS) in organisms which interact with critical macromolecules, such as DNA, proteins or membranes and lead to mutations and chromosomal aberrations [14]. The DNA damage is one of the most important side effects of ionizing radiation [15]. The ROS mediated bimolecular reactions and their relationship with radiation side effects are the current subject of scientific investigations in radiotherapy. To address this problem, some chemical agents have been investigated as scavengers of ROS, thus serve as effective radioprotectors againstzradiation damage [16]. Another benefit is their supplementation of antioxidants to improve the efficacy of radiotherapy. A wide range of phytochemicals that have been investigated for this purpose include, flavonoids, polyphenols, carotenoids, and organosulfur compounds [17].

**Fig. 1:**
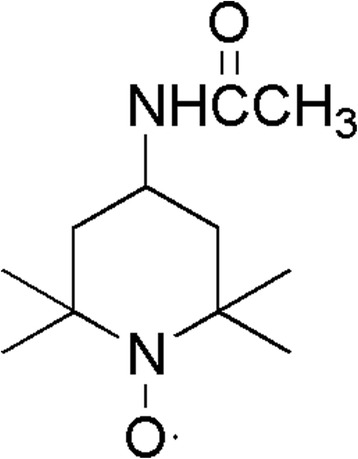
The chemical structure of AA-Tempol

In recent years, nitronyl nitroxide radicals have been extensively studied due to their antioxidative properties to protect against oxidative damage [18–20]. Nitronyl nitroxide radicals can directly react with ROS [21] to prevent the reduction of hydrogen peroxide to the hydroxyl radical [22], which occured through the redox transformations between the oxidation states of nitroxide, hydroxylamine, and the oxoammonium cation. The nitroxide and oxoammonium cation paired the redox couple. The redox couple which paired by nitroxide and oxoammonium cation can support catalytic processes via reversible one-electron redox reactions. The hydroxylamine can function as an efficient hydrogen atom donor and provide antioxidant defense [23]. Tempol (4-hydroxy-2,2,6,6-tetramethylpiperidine-N-oxyl) represents this new family of nitroxides that act as an effective antioxidant in scavenging superoxide anions which has been shown to have protective effects against ionizing radiation [24, 25]. Tempol has been shown to protects mammalian cells against radiation-induced cytotoxicity in vitro [26] and can afford protection in vivo against whole- body irradiation [27]. It was reported that a mitochondria-targeted nitroxide/hemigramicidin S conjugate (TPEY-Tempol) protected mouse embryonic cells against gamma irradiation significantly [28, 29]. To search for more effective radiation protectors with minimal toxicity, a water-soluble nitroxides Acetamido-Tempol (AA-Tempol, Fig. 1) was evaluated for potential radioprotective properties using HUVEC cells and clonogenic assays. Its radioprotective effects in vitro were evaluated in this study. The results showed that AA-Tempol increased the level of GSH and decreasing the level of MDA which acted as antioxidants by scanvenging ROS. The present data also showed that pretreatment with AA-Tempol can attenuated X-radition induced cell apoptosis by regulating the expression levels of Bcl-2, Bax, caspase-3 and P53. These observations indicate that AA-Tempol may be a potential therapeutic agent against the radiation damage.

## Material and methods

### Chemicals

4-Acetamido-2,2,6,6-tetramethylpiperidin-1-oxyl was purchased from Sigma (Beijing, China). All other chemical reagents were purchased from J&K Scientific Ltd. (Beijing, China), and other chemicals and reagents were purchased from Sigma (Beijing, China), unless otherwise indicated. All chemicals and reagents used were commercially available and of standard biochemical quality.

### Cells

The Human Umbilical Vein Endothelial cell line (HUVEC) was provided by the Department of Preventive Medicine, the Fourth Military Medical University (No. 169, Changle West Road, Xi’an, Shaanxi 710032, PR China). They were grown at 37 °C in a humidity of 5% CO_2_ in Dulbecco’s modified Eagle’s medium (DMEM) supplemented with 10% fetal bovine serum, 10000 units/mL penicillin and 50 μg/mL streptomycin.

### Cytotoxicity assay

The cytotoxicity assays was performed by the Cell Counting Kit-8 (CCK-8) method [30]. HUVEC cells were seeded into 96-well plates at a density of 2 × 10^4^ cells per well and incubated at 37 °C in a humidified atmosphere with 5% CO_2_ for 24 h. The medium was removed and fresh DMEM containing the appropriate dilution of the AA-Tempol was added. AA-Tempol were dissolved in 0.01 mol/L Phosphate Buffered Saline(PBS, pH 7.4) at 32 mmol/L. HUVEC cells were seeded into 3 96-well plates at a density of 2 × 10^4^ cells per well and incubated at 37 °C in a humidified atmosphere with 5% CO_2_ for 24 h. Then the medium was removed, the AA-Tempol solution and fresh DMEM were added at a final AA-Tempol concentration of 0, 0.0625, 0.25, 1, 2, 4, 8, 16 mmol/L respectively. Each concentration has 5 duplicate wells in one plates. After treatment for 0.5, 1 and 2 h respectively, HUVEC cells in the plates were cultured for another 48 h in fresh DMEM, then treated with Cell Counting Kit-8 reagent (10 μL/ well) for 2 h at 37 °C. The absorbance was read at 450 nm. The experiments were performed at least three times.

### colony survival assay

Colony survival assay was performed using Giemsa staining method as previously described [31]. Cells were seeded onto 6-well plates and allowed to grow for 24 h prior to treatment with different concentrations of the AA-Tempol. AA-Tempol was solubilised with 0.01 mol/L PBS and diluted in culture media to achieve concentrations of 0, 1 and 2 mM. Control cells were prepared in a similar way except that the cell was not pretreated with AA-Tempol before irradiation. Irradiation (4 and 6 Gy) was performed with a X-ray machine at dose rate of 4.404Gy/min. The serial dilution of AA-Tempol with DMEM media in cultured plates yielded final concentrations of 0, 1, 2 mM. The different concentrations of AA Tempol was added onto the cells at 1 h before X-ray irradiation (0Gy,4Gy,6Gy). After 10 to 14 days incubation in fresh DMEM in a humidified atmosphere containing 5% CO_2_, the plates were fixed with 70% MeOH and stained with 0.8% Swiss Giemsa. And colonies which come from an original surviving cell of 50 or more cells were counted as survivors. The surviving fractions were calculated as the plating efficiency of samples relative to that of sham-irradiated control. The survival fractions were calculated as (number of colonies/number of cells plated)/(number of colonies for corresponding sham-irradiated control/number of cells plated) [32]. The experiment was performed at least six times.

### Hoechst staining

Cell death was determined by Hoechst 33258 (Sigma) fluorescent staining. Cell suspensions were seeded into 24-well plates at a concentration of 2 × 10^4^ cells per well. Cells were allowed to attach for 24 h; and then treated with 2 mM AA-Tempol for 1 h before x-radiation (0 and 8 Gy). The cells were washed with PBS for three times at 0.5 h after X-ray irradiation and then incubated for 48 h with DMEM. Then the cells were stained with Hoechst 33258 (10 μg/ml) for 15 min, and then fixed by 4% paraformaldehyde for 10 min. Cells were observed under a fluorescence microscope (Olympus BX61, Japan). The Hoechst dye were excited at 340 nm. For each well, three visual fields were selected randomly.

### Flow cytometric (FCM) analysis of the cell apoptosis

Apoptosis analyses were performed as described previously [33]. Briefly, apoptosis and cell viability were measured by using annexin V-FITC (Assay Designs, Inc. USA) and PI double staining. HUVEC cells were seeded into 10 cm plates at a density of 5 × 10^5^ cells per well and allowed to grow for 24 h prior to treatment with AA-Tempol. Then AA-Tempol was added to cultured cells maintaining the final concentration of 1.0 mmol⋅L^−1^ for 1 h before 8 Gy X-radiation. At 0.5 h after radiation, the medium was removed and fresh DMEM was added onto HUVEC cells. The untreated cells (blank control) as well as the treated cells were incubated for 4 days. Then cells were harvested and washed twice with 0.01 mol/L PBS. Before they were measured by flow cytometer, ethanol was washed off with PBS. The cells were diluted in 100 μl of 1× annexin-binding buffer per assay and incubated with annexin V-FITC and PI for 15 min at room temperature in the dark. Having been washed with PBS, they were measured by Epics XL-4 flow cytometer (BECKMAN-Coulter, USA) using Cellquest software (version2) to obtain the information on apoptosis.

### Western blot analysis

Western blot analysis was performed as described previously [34]. Cultured cells were preincubated with the concentrations of AA-Tempol (1.0 mmol⋅L^−1^) at 37 °C for 1 h, then cells were exposed to 8 Gy X-radiation and cultured for 48 h in fresh DMEM. Protein concentrations were determined by using the BCA assay kit. Proteins of total extracts were separated on 10% SDS gels. Separated proteins were transferred electrophoretically onto polyvinylidene difluoride membrane (PVDF) (Amersham International) and blocked with 5% non-fat milk in phosphate buffered saline (PBS)-Tween for 2 h. The primary antibodies polyclonal rabbit anti-Bcl-2 (dilution ratio 1:2000), anti-p53 (dilution ratio 1:2000), anti- caspase3 (dilution ratio 1:3000), GAPDH (1:5000) and with β-actin (dilution ratio 1:3000) were incubated with the membranes at 4 °C overnight. The membranes were incubated with a secondary antibody conjugated to horseradish-peroxidase (Beyotime) after being washed with TBST three times for 10 min each time, and bands were visualized using an ECL system (Perkin Elmer). Semi-quantitation of scanned films was performed using Quantity One-4.6.2 (Bio-Rad, Italy). The relative expression levels of the targeted proteins were determined using beta-actin as a loading control. All western blots were independently replicated three times.

### Biochemical assays

Cultured cells were pretreated with varied concentrations of AA-Tempol (0, 0.25, 1, 4 mmol⋅L^−1^) at 37 °C for 1 h before 8 Gy X-radiation and cultured for another 48 h in fresh DMEM, then cells were harvested and suspended in 0.2 ml PBS containing 0.1 ml RIPA lysis buffer (50 mM Tris, 150 mM NaCl, 1% Triton X-100, 1% sodium deoxycholate, 0.1% SDS, sodium orthovanadate, sodium fluoride, EDTA, leupeptin, 1 mM PMSF) and intermittently vortexed for 10 min (4 °C) and centrifuged at 12,000 × rpm for 10 min (4 °C). The supernatant was taken for biochemical estimations. The level of lipid peroxidation (MDA) was assayed by analyzing TBA-reactive substances which were determined by a method utilizing the lipid peroxidation assay kit. The pink-colored chromogen formed by the reaction of TBA with breakdown products of lipid peroxidation was measured [35]. The contents of reduced glutathione (GSH) were assayed by the commercially available cellular GSH assay kit. In each group, six samples (*n* = 6) were processed. The cell protein was determined by the Lowry method using BSA as standard. The experiment was performed at least six times.

### Statistical analysis

The statistical analysis of the data to determine significant variations between the groups was performed using SPSS statistical software. The one-way analysis of variance (ANOVA) test was performed, and *post hoc* multiple comparisons were performed using the least significant difference (LSD) test. The results are presented as the means ± standard deviation (SD). In all cases, *P <* 0.05 was considered to indicate a statistically significant difference.

## Results

### Cytotoxicity and clonogenic protection of AA-Tempol

**Fig. 2:**
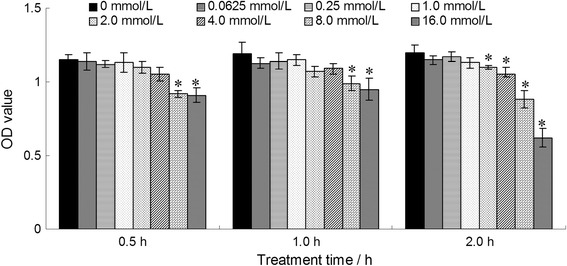
The cytotoxicity of different concentrations (0, 0.0625, 0.25, 1, 2, 4, 8 and 16 mmol⋅L^−1^) of AA-Tempol exposure for 0.5, 1, and 2 h on HUVEC cells was examined by CCK-8 assay. The cells treated with 0.0625, 0.25, 1, 2, 4 mmol⋅L^−1^ AA-Tempol for 0.5 and 1 h did not displayed cell growth inhibition. The cells treated with 8 and 16 mmol⋅L^−1^ AA-Tempol exposure for 0.5 and 1 h and 2, 4, 8 and 16 mmol⋅L^−1^ AA-Tempol exposure for 2 h displayed mild cell growth inhibition. *P < 0.05 vs. normal control group

The cytotoxicity of AA-Tempol on HUVEC cells was examined using the CCK-8 assay. As shown in Fig. 2, AA-Tempol did not inhibit cell growth under the concentration of 4 mM when exposed for 0.5 h and 1 h. But when treated with the concentration of 8 and 16 mM AA-Tempol for 0.5 and 1 h, the optical density (OD) value of HUVEC cells decreased dramatically which indicated that the higher concentrations of AA-Tempol inhibited the cell growth greater than those with lower concentration (*P <* 0.05). When exposure for 2 h in 2, 4, 8 and 16 mmol/L AA-Tempol, the HUVEC cells growth were significantly inhibited (*P <* 0.05). Therefore, the radioprotection effects of being exposed for 1 h in 0.25, 1, 2, 4 mmol/L AA-Tempol were investigate on HUVEC cells.

**Fig. 3:**
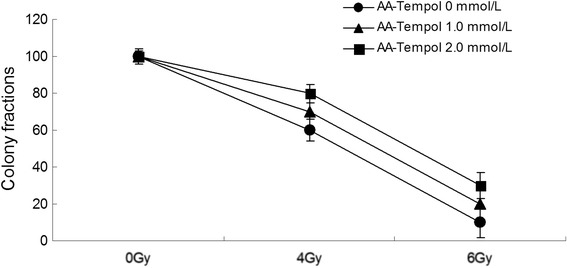
The radioprotective effect of AA-Tempol on the viability of irradiated HUVEC cells was examined using clonogenic survival assay. Pretreatment of different concentrations (0, 1 and 2 mmol⋅L^−1^) of AA-Tempol 1 h prior to X-radiation provided significant protection compared to cells treated with radiation alone (*P <* 0.05)

To study the anti-radiation effect of AA-Tempol in cell culture, we examined the viability of irradiated HUVEC cells using a clonogenic survival assay. As shown in Fig. 3, pretreatment with AA-Tempol 1 h before X-radiation increased the colony survival (*P <* 0.05) significantly compared with the cells treated with radiation alone. The maximum protection was achieved at 2.0 mmol⋅L^−1^ AA-Tempol with radiation at 4 Gy (*P <* 0.05).

### Protective effects against apoptosis of AA-Tempol

**Fig. 4:**
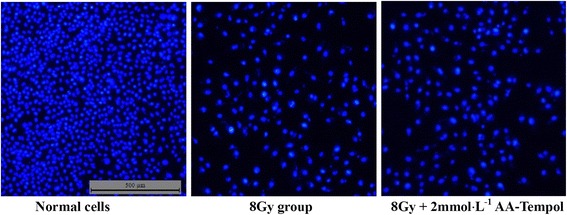
Hoechst 33258 and PI staining in cultured HUVEC cells. Representative fluorescence images obtained after Hoechst 33258 staining in Normal group, 8Gy X-radiation treated group, 8Gy X-radiation + 2 mmol⋅L^−1^ AA-Tempol treated group

**Fig. 5:**
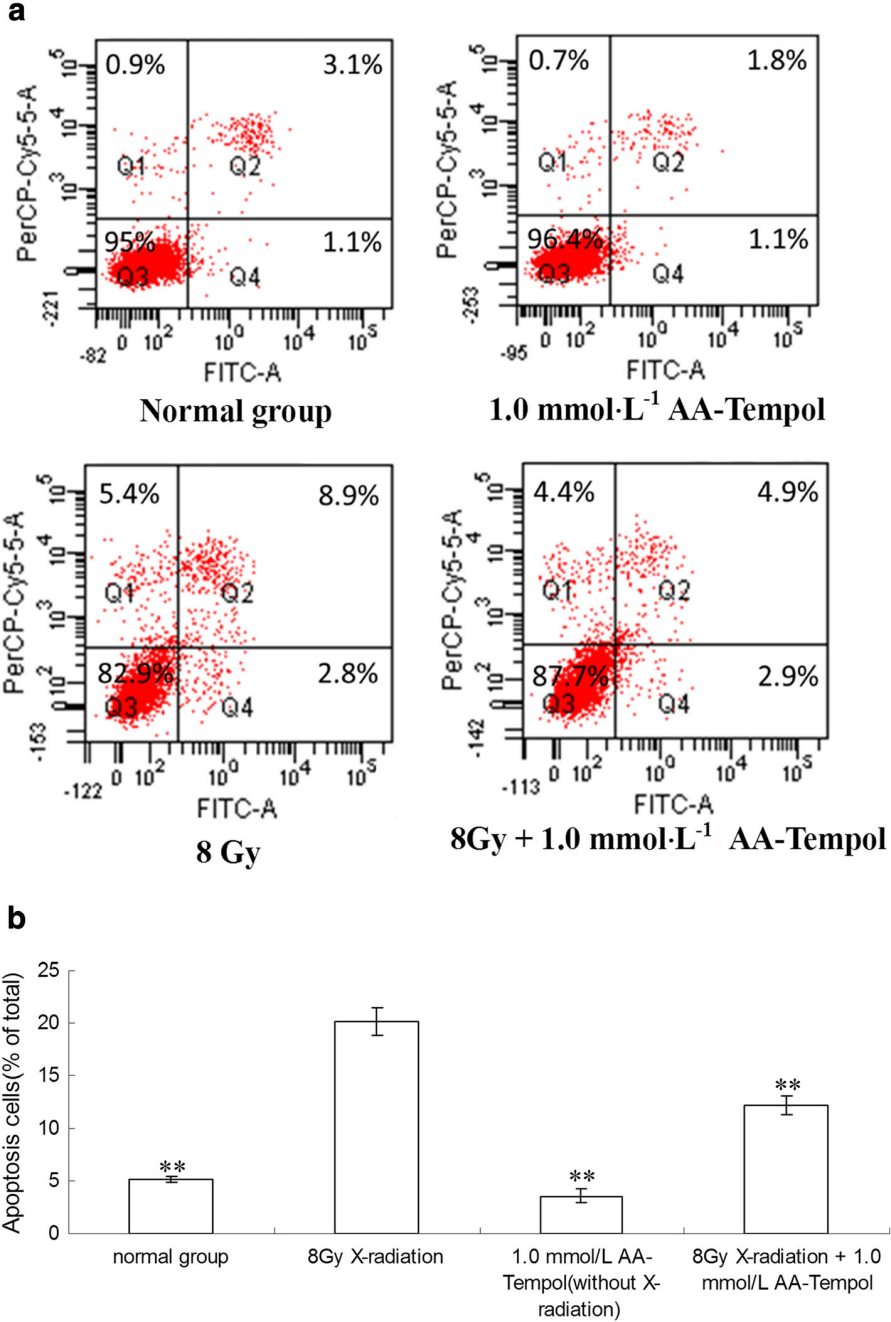
The anti-apoptosis effect of different concentrations (0, 0.25, 1, 4 mmol⋅L^−1^) of AA-Tempol pretreatment for 1 h was analyzed using Annexin V/propidium iodide staining in a flow cytometry assay. **a** AA-Tempol pretreatment attenuated radiation-induced apoptosis in HUVEC cells. **b** The bar graph of apoptotic cells expressed as a percentage of total cells for each treatment with SAA from six experiments. Data are presented as mean ± S.D. (*n* = 6). ** *P <* 0.01 vs. 8 Gy group. Q1, Q2, Q3 and Q4 represent dead, late apoptosis, vital and early apoptosis cells, respectively

We evaluated the anti-apoptosis effects of AA-Tempol on HUVEC cells using Annexin V/propidium iodide staining in a flow cytometry assay. The results showed that X-Ray radiation dramatically increased the number of apoptotic cells compared with the normal control group. Pretreatment of HUVEc cells with AA-Tempol pretreatment significantly reduced the radiation-induced apoptosis in HUVEC cells compared to the 8Gy X-radiated cells (*P <* 0.01) (Figs. 4 and 5). This implies that AA-Tempol demonstrated a protective effect in irradiated HUVEC cells, thus reducing apoptosis from 20.1 ± 1.3% in 8 Gy X-radiated cells in untreated or control cells to 12.2 ± 0.9% in cells treated with 1 mM AA-Tempol). This suggests that the radioprotective effect of AA-Tempol in HUVEC cells was due to the attenuation of radiation-induced apoptosis.

### AA-Tempol upregulated Bcl-2 expression and downregulated P53, Bax and caspase-3 expression

**Fig. 6:**
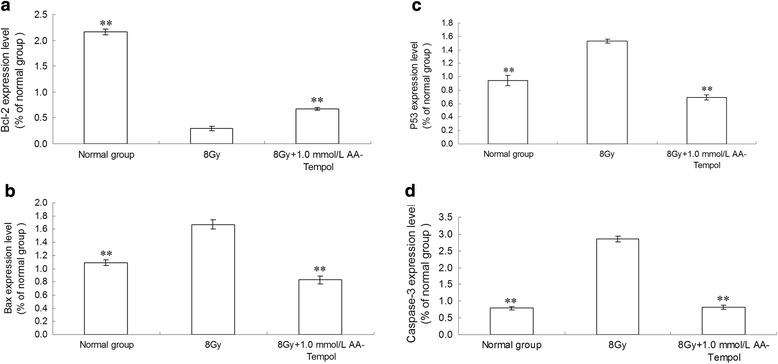
The effect of AA-Tempol on the protein expressions of Bax, Bcl-2, P53 and caspase-3 in HUVEC cells was determine by immunoblotting assay (**a**, **b**, **c**, **d**) Semi-quantified data for the level of the proteins are shown in the lower panel (**e**). Data represent mean ± S.D. (*n* = 6). **P < 0.01 vs. 8 Gy group

To elucidate the molecular mechanism of the anti-apoptosis effect of AA-Tempol against X-radiation induced HUVEC cell apoptosis, the expression of Bax, Bcl-2 and p53 and caspase-3 were examined as shown in Fig. 6. The results showed that X-radiation induced a sharp increase in P53, Bax and the active fragment of caspase 3 expression and a marked decrease in Bcl-2 expression. Compared with the control group, AA-Tempol treatment can significantly block the increase of caspase-3 activity in radiated HUVEC cells (*P <* 0.01). The expressions of Bax and P53 were also down-regulated and the expression of Bcl-2 was up-regulated in the 1.0 mM AA-Tempol pretreatment groups.

### Biochemical markers

**Fig. 7:**
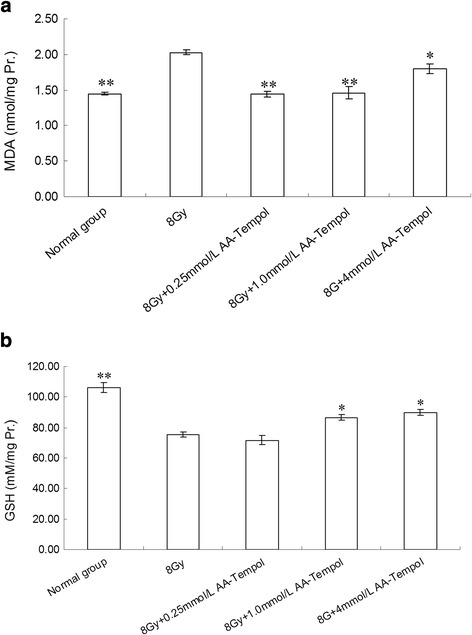
The effect of AA-tempol on the activity of the level of MDA (**a**) and GSH (**b**) in HUVEC cells *P < 0.05, **P < 0.01 vs. normal group

The changes in the level of malondialdehyde (MDA) and glutathione (GSH) in HUVEC cells after X-radiation are presented in Fig. 7. The results showed that X-radiation caused a dramatic decrease in GSH level and a significantly increase in MDA activity compared to the normal control group (*P <* 0.01). Pretreatment with AA-Tempol can decrease MDA activities (*P <* 0.01) and increase the GSH level (*P <* 0.05) in HUVEC cells significantly compared to the 8Gy X-radiated cells.

## Discussion

The present study was to evaluate the effects of X-radiation on the antioxidant defence system and the radioprotection afforded by AA-Temopl. The radiation energy causes damage to living tissues through a series of molecular events depending on developing reactive oxygen species (ROS) which are generated by the action of radiation on water. The major free radicals formed upon aqueous radiolysis are hydroxyl radical (⋅OH) and superoxide radical (O^⋅2-^) [36]. It has been reported that ionising radiation at cellular level induces damage in the biologically important macromolecules such as proteins, lipids, as well as nucleic acids [37]. Approximately 65% of the DNA damage is caused by the indirect effect of these free radicals [38] and trigger the oxidation of biomolecules leading to the formation of various secondary free radicals, which can chemically modify DNA, proteins, and lipids, resulting in further cellular damage [39]. Therefore, antioxidants potentially provide protection from radiation.

It was reported that Tempol-treated TK6 human lymphoblastoid cells, which were known to undergo apoptosis in response to radiation exposure, demonstrated a decrease in cytotoxicity after exposure to a 6-Gy dose of ionizing radiation [40]. In a related experiment, nitroxides were found to protect against lipid peroxidation [41]. An early radioprotection study showed that the administration of Tempol to Chinese hamster cells exposed in culture to lethal doses of gamma-radiation resulted in a significant and dose-dependent protective effect with a protection factor of 2.5 compared to the untreated cells. Stephen M. Hahn reported two Tempol derivatives, Tempamine and 3-aminomethyl-PROXYL, exhibited greater radioprotection than Tempol [42]. A phase I clinical trial in patients receiving whole brain radiotherapy suggested that Tempol may be effective at preventing radiation-induced alopecia with only mild (grade I and II) toxicity [43].

It is known that GSH plays an important role in the antioxidation of reactive oxygen species (ROS) and free radicals [44–46]. It is a potent free radical and reactive oxygen species scavenger [47]. Irreversible cell damage supervenes when the cell is no longer able to maintain its GSH content [48]. In the present study, we found that X-radiation caused a dramatic decrease in the level of GSH and an increase in the activity of MDA in HUVEC cells compared with the normal group (*P <* 0.01), which showed that X-radiation can damage the redox balance. It has been reported Tempol can increased cell survival and protected against DNA damage induced by the mutagen neocarzinostatin (NCS), which had previously been shown to induce its mutagenic activity *via* a GSH-dependent mechanism [49]. We found that when pretreatment with AA-Tempol, a derivative of Tempol, significantly decreased the activitiy of MDA and increased the GSH level in HUVEC cells compared with the control group (without AA-Tempol pretreatment, *P <* 0.05). This confirms that AA-Tempol provids protective effects against X-radiation damage through mediate the antioxidation system in vitro.

Induction of apoptosis by ionizing radiation is one of the mechanisms implicated in radiation-induced cytotoxicity. During apoptosis induction, many different activation cascades eventually lead to caspase-3 cleavage and subsequent cell death [50]. For instance, our previous studies have revealed that radiation induced apoptosis by the release of mitochondrial cytochrome C, increasing the activation of caspase-3, and up regulating the expression of Bax and P53 and down regulating the expression of Bcl-2 [51]. Proteins in the Bcl-2 protein family are important in the regulation of cell apoptosis. Bcl-2 prevents the opening of the mitochondrial membrane pores and is the most important anti-apoptotic gene [52], whereas Bax induces the opening of membrane pores and is a very important pro-apoptotic gene [53]. Under the oxidative stress, the transcription factor p53 is activated and stabilized. Subsequently, p53 up regulates the expression of genes that facilitate apoptosis, DNA repair or genomic stability [54]. In the present study, the results showed that X-radiation dramatically increased the numbers of apoptotic cells and induced a sharp increase in the Bax, caspase-3 and P53 expression levels and a marked decrease in the Bcl-2 expression level compared with the normal control group. The cell apoptotic death in by radiation is associated with the elevated production of ROS. Supplementation of antioxidants to improve the efficacy of radiotherapy is today’s proposed strategy [55]. For instance, Samuni AM found that Tempol could prevent cell death in lymphoblastoid cells, which undergo apoptosis in response to radiation exposure and inhibit a radiation-induced increase in p53 observed in untreated control cells [40]. Our research indicated that in HUVEC cells, preatrement of AA-Tempol up-regulated the expression of Bcl-2 and down-regulated the expressions of Bax, caspase-3 and P53 after X-radiation exposure. The ability of AA-Tempol to attenuate cell apoptosis and regulate the expression levels of Bcl-2, Bax, caspase-3 and P53 may be due to its antioxidative capacity, which prevents the accumulation of ROS and other toxic materials to prevents the induction of cell death.

We believe that the mechanism of AA-Tempol to protect against ionizing radiation may due to its unique ability to scavenge the ROS. AA-Tempol belongs to nitroxides, are chemical compounds containing the tertiary amine (R_3_N^+^-O^−^) functional group that are oxidized to form relatively stable nitroxide radicals. These compounds are membrane-permeable radical scavengers which have SOD and catalase activities though an oxoammonium/nitroxide redox couple [56–58]. Firstly, nitroxide radical RR’NO⋅ is converted to the oxoammonium cation (RR’NO^+^) by the oxidation of protonated form of superoxide (HO_2_⋅) [59]. Then RR’NO^+^ can be reduced by O_2_^⋅−^ back to the nitroxide radical.

RR’NO⋅ + O_2_^⋅−^ + 2H+ → [RR’NO^+^] + H_2_O_2_

[RR’NO^+^] + O_2_^⋅−^ → RR’NO⋅ + O_2_

In the whole process, the nitroxide acts as a catalyst and is not consumed in the process of dismutation of O_2_^⋅−^ to H_2_O_2_ and oxygen. Furthermore, nitroxides and hydroxylamines can inhibit lipid peroxidation by participating in redox reactions at every step [60]. Additionally, nitroxides were shown to confer catalase-like behavior to heme proteins and to detoxify H_2_O_2_ [61] and to participate in radical-radical recombination reactions, which can limit the levels of free radicals and protect cells [62].

In conclusion, the protective effect of AA-Tempol for the antioxidant defenses in HUVEC cells was investigated. The results indicated that AA-Tempol increased the level of GSH and decreasing the level of MDA which acted as antioxidants by scanvenging ROS. The present data also showed that pretreatment with AA-Tempol can attenuated X-radition induced cell apoptosis by regulating the expression levels of Bcl-2, Bax, caspase-3 and P53. These observations indicate that AA-Tempol may be a potential therapeutic agent against the radiation damage.
